# Small extracellular vesicles as a multicomponent biomarker platform in urinary tract carcinomas

**DOI:** 10.3389/fmolb.2022.916666

**Published:** 2022-09-27

**Authors:** Szeliski K, Drewa T, Pokrywczyńska M

**Affiliations:** Department of Regenerative Medicine, Cell and Tissue Bank, Chair of Urology and Andrology, Collegium Medicum, Nicolaus Copernicus University, Bydgoszcz, Poland

**Keywords:** extracellular vesicles (EVs), prostate cancer, urinary bladder cancer (UBC), renal cell carcinoma (RCC), cancer diagnosis, small extracellular vesicles (sEVs)

## Abstract

Extracellular vesicles are a large group of nano-sized vesicles released by all cells. The variety of possible cargo (mRNAs, miRNAs, lncRNAs, proteins, and lipids) and the presence of surface proteins, signaling molecules, and receptor ligands make them a rich source of biomarkers for malignancy diagnosis. One of the groups gathering the most interest in cancer diagnostic applications is small extracellular vesicles (sEVs), with ≤200 nm diameter, mainly composed of exosomes. Many studies were conducted recently, evaluating the diagnostic potential of sEVs in urinary tract carcinomas (UTCs), discovering and clinically evaluating various classes of biomarkers. The amount of research concerning different types of UTCs understandably reflects their incidence. sEV cargos getting the most interest are non-coding RNAs (miRNA and lncRNA). However, implementation of other approaches such as metabolomic and proteomic analysis is also evaluated. The results of many studies indicate that sEVs have an essential role in the cancer process and possess many possible diagnostic and prognostic applications for UTC. The relative ease of obtaining biofluids rich in sEVs (urine and blood) confirms that sEVs are essential for UTC detection in the liquid biopsy approach. A noticeable rise in research quality is observed as more researchers are aware of the research standardization necessity, which is essential for considering the clinical application of their findings.

## 1 Introduction

### 1.1 Urinary tract carcinomas

The urinary tract carcinomas (UTCs) make a group of the most common types of cancer, right after lung carcinomas. Considering both genders in 2020, the incidence of prostate cancer was the third, urinary bladder 11th, and kidney 15th with 1,414,259; 573,278; and 431,288 newly diagnosed cases, respectively. Despite many efforts, one of the main consistent problems is proper and early diagnosis, with possibly the least invasive methods. The death numbers of these cancer types constitute 27, 37, and 42% of newly diagnosed prostate, urinary bladder, and kidney cases, respectively (Cancer, 2020). The lack of efficient and non-invasive diagnosing tools results in a high level of over diagnosis and unnecessary biopsies, with no benefits in treatment, especially in prostate cancer, (Hayes and Barry, 2014).

### 1.2 Extracellular vesicles

**FIGURE 1:**
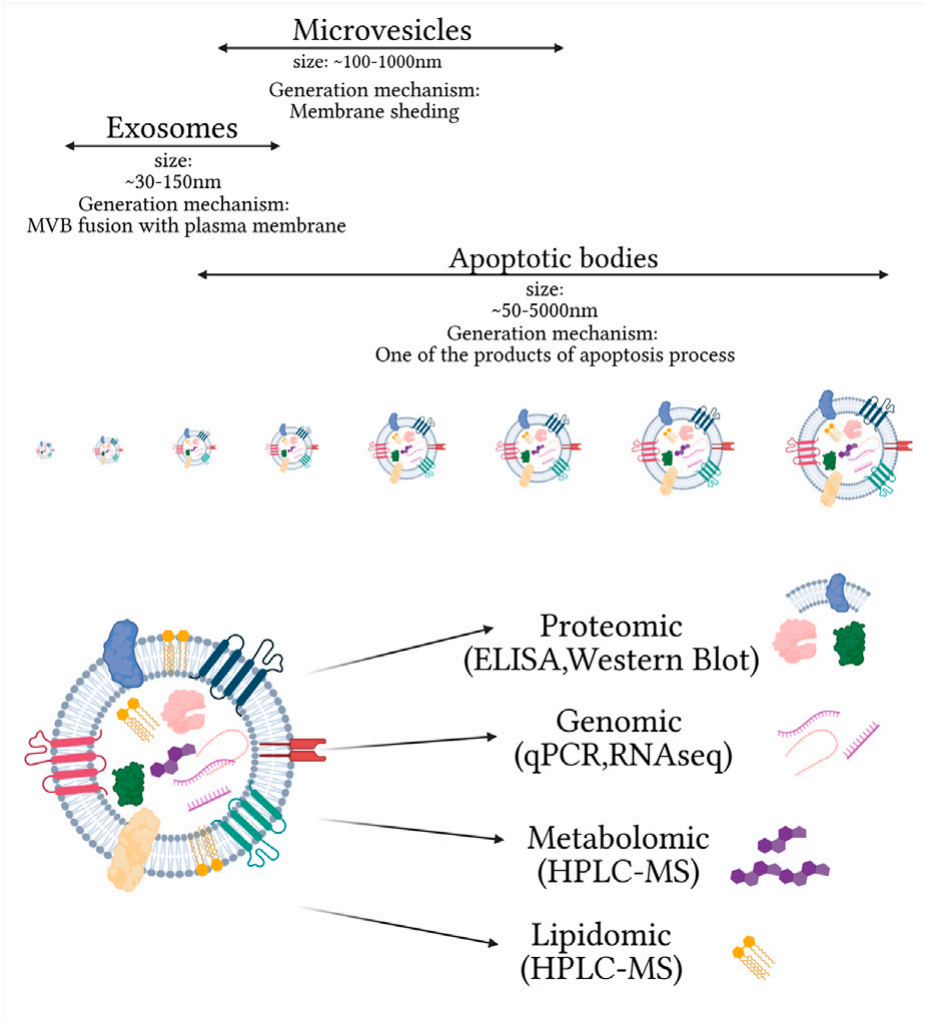
Main extracellular vesicle subtypes and complexity of small extracellular vesicle composition, on omics used for their analysis. Created with biorender.com.

Extracellular vesicles (EVs) are a heterogeneous group of membranous vesicles released by all types of cells. They differ in size, biogenesis, release mechanisms, cargo (mRNAs, miRNAs, lncRNAs, and proteins), and subcellular markers (Mathieu et al., 2019). Their presence has been confirmed in many biofluids such as blood, urine, saliva, cerebrospinal fluid, and others (Arraud et al., 2014; Iwai et al., 2016; Muraoka et al., 2020; Musante et al., 2020). The role of EVs in different pathomechanisms, especially by the cargo they are carrying, caused a rise in interest in their potential application as a multicomponent biomarker platform in clinical diagnosis (Figure 1). One of the main factors determining the classification of EVs is their biogenesis mechanism. Based on this criterium, three main categories of EVs can be distinguished: apoptotic bodies, microvesicles, and exosomes. Apoptotic bodies (ABs) are generated within programmed cell death and are characterized by the biggest size among EVs, ranging from ∼50 to 5000 nm (Battistelli and Falcieri, 2020). Microvesicles (MVs) are a class of EVs with a smaller size range than ABs ranging within 100–1000 nm. Their primary mechanism of generation is based on outward budding and fission of the plasma membrane. Many cell-dependent factors, including membrane composition, are incorporated in this mechanism as repositioning of the outer and inner parts of phosphatidylserine and redistribution of phospholipids are observed. The main pathways inducing the release of MVs are 1) ARRDC1, TSG101, and VSP4 dependent; 2) hypoxia following the expression of RAB22A via HIF, and 3) ARF6, PLD, ERK, and MLCK cascade (Abels and Breakefield, 2016). Based on their biogenesis, the third class of EVs is exosomes with the smallest range of size ∼30–150 nm. Their biogenesis is mostly explored among EV subtypes and is based on intraluminal vesicle (ILV) formation within multivesicular bodies (MVBs). The formation of ILVs is associated with endosome membrane reorganization and tetraspanins enrichment. During this process, cargo is packed within newly formed vesicles with endosomal sorting complex (ESCRT) dependent and ESCRT independent pathways. The ESCRT-dependent pathway is based on detecting specifically ubiquitinated proteins and selection via interaction with syndecan (Villarroya-Beltri et al., 2014). The ESCRT-independent pathway is associated with raft-based microdomains enriched in sphingomyelinases and ceramide formation. In this mechanism, tetraspanins, enriched during the formation of ILVs and other specialized mechanisms, are also involved (Abels and Breakefield, 2016; Zhang Y. et al., 2019).

However, consensus about specific markers of different subtypes of EVs has not yet emerged, as they might be highly cell dependent. For example, red blood cell–derived exosomes do not present characteristically for this class of EV tetraspanins on their surface (Kuo et al., 2017). A similar problem is met with purification methods, which at this moment are thought to never fully separate just one pure subtype of EVs (Thery et al., 2018). Thus in this study, the small extracellular vesicles (sEVs) will be considered: EVs of diameter ≤200 nm and presenting on their surface at least one of the following markers: CD9, CD63, and CD81, referred to by many researchers as exosomes.

### 1.3 Extracellular vesicles in malignancies

**FIGURE 2:**
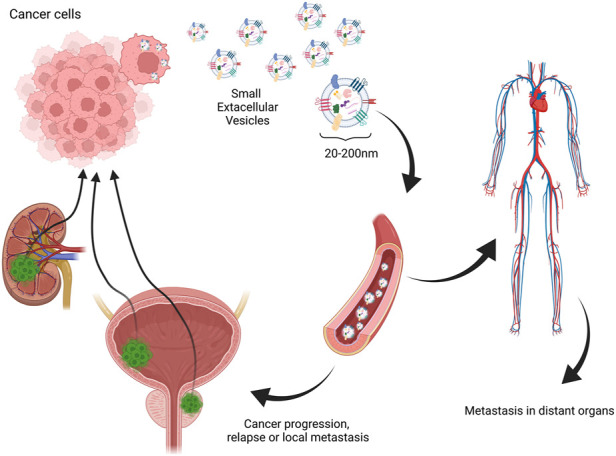
One of the hypothesized mechanisms of extracellular vesicle involvement in relapse, local, and distant metastasis in urinary tract carcinomas. Created with biorender.com.

Many researchers have evaluated the role of EVs in pathomechanisms of carcinomas. One of the leading hypotheses is that EVs, mainly small EVs such as exosomes released by cancer cells, contain genetic material sufficient to cause metastasis niche when integrated with normal cells (Fujita et al., 2016). Another important aspect is the involvement of EVs in the promotion of angiogenesis. As sEVs are already confirmed to carry angiogenesis-inducing vascular endothelial growth factor, which not only might promote vascularization in the tumor site, but also activate epithelial–mesenchymal transition (EMT) (Hida et al., 2018; Li C. Y. et al., 2019). Another significant involvement of EVs in malignancy development is their role in immunomodulation and immune evasion. Different studies have shown that different cancer-derived EVs may carry higher levels of immunosuppressive molecules such as macrophage migration inhibitory factor or PD-L1 forming metastatic niche (Costa-Silva et al., 2015; Chen et al., 2018; Ricklefs et al., 2018). This mechanism could be responsible for local transmission and relapses and considering the ease of EV migration to the main blood circuit and distant metastasis (Figure 2). These facts encourage analysis of EVs in cancer patients for diagnostic procedures and finding new diagnostic targets. However, many technical difficulties are met considering one of the first steps—the isolation procedure. Several studies show that the choice of method plays a vital role in further downstream analysis (Thery et al., 2018). The difficulty of repetitive, time- and cost-efficient isolation procedures is one of the most significant drawbacks of the possible clinical application of EVs. Nevertheless, it is a challenge to face before their potential application in routine diagnostic procedures.

## 2 Materials and methods

**FIGURE 3:**
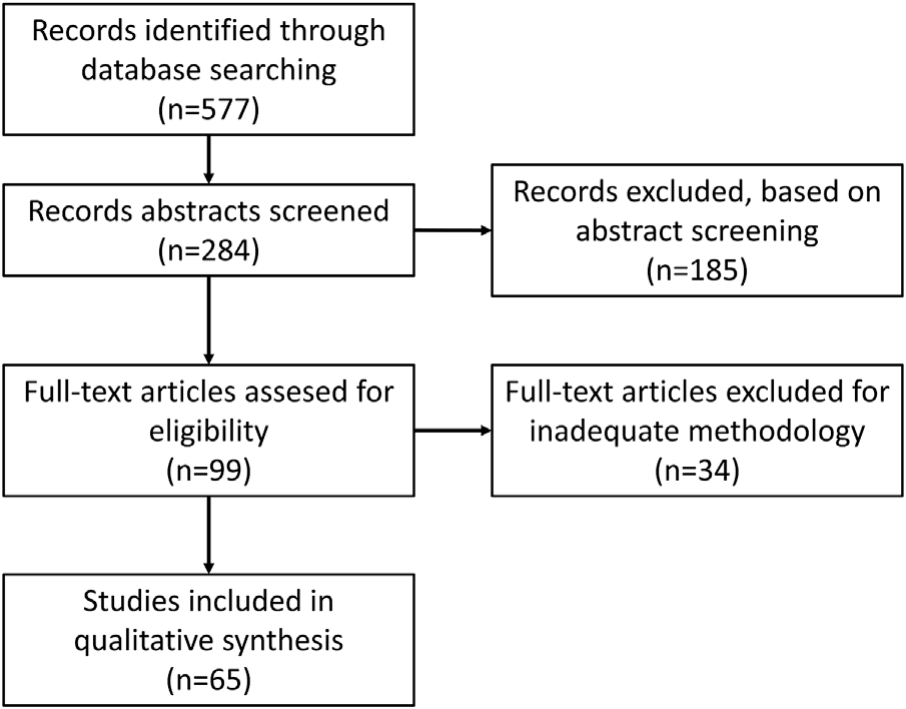
Literature screening flow diagram.

To present the most up-to-date research situation and current trends in this field, publications from 2017 to February 2022 were chosen. The PubMed database was chosen for searching articles. The keywords combinations selected for search: “prostate”/“urinary bladder”/“renal” + “cancer” + “exosomes”/“extracellular vesicles” + “diagnosis”. A total of 284 articles were found. A total of 185 of them were excluded during abstract screening as they were review articles or were not connected with the subject of this study. Additionally, 34 studies were disqualified for the inaccurate or unclear description of sEV purification/concentration methods or lack of quantification, size or characteristic sEV marker analysis, and procedures that were necessary to properly analyze the material of sEV origin when not performed by outside facilities (Figure 3).

## 3 Potential role of sEVs as biomarkers for different UTC subtypes

### 3.1 Prostate cancer

Prostate cancer (PCa)–specific sEV usage approaches are among the most evaluated possible diagnostic applications of sEVs. Most of the research in this field focuses on miRNAs (microRNAs) and proteins carried by sEVs, as it was proven that most cancer-associated DNA is carried rather by larger vesicles than that by sEVs (Vagner et al., 2018). Moreover, the necessity for distinct analysis of the different sizes of EVs was highlighted by the results of Lazaro-Ibanez et al. (2017)where researchers showed that different EV classes carry distinctly distributed cargos (Lazaro-Ibanez et al., 2017). However, an approach not related to sEVs carried cargo but rather physical properties of sEVs from PCa patients were also proposed. In a recent study, Logozzi et al. (2021) performed a comparative analysis of the size and concentration in plasma sEVs of PCa patients and healthy control. The study results show a significantly higher concentration of sEVs with an average smaller size in PCa patients, with a sensitivity of 89% and specificity of 71% in distinguishing from a healthy control. However, these results were obtained with just one of the methods for size and concentration analysis (NTA), and thus should be confirmed with a broader use of analysis methods (Logozzi et al., 2021).

#### 3.1.1 miRNA and lncRNA

Non-coding RNAs, including microRNAs and long non–coding RNAs, are among the most explored types of sEV cargos in PCa diagnosis. One of the facts encouraging exploring these types of molecules is that the PCA3 test for PCa diagnosis is the only FDA-approved method utilizing non-coding RNA for cancer detection (De La Taille et al., 2011). In many studies, this marker alone has been proven more accurate than standard prostate-specific antigen (Luo et al., 2014). The approach for analysis of this clinically proven biomarker for specific sEV analysis was performed by McKiernan et al. (2020)where urinary sEV PCA3, ERG, and SPDEF RNAs were evaluated in previously negative biopsy patients. Compared to other non-invasive methods, this panel is superior to PSA alone and the European Randomized Study of Screening for Prostate Cancer Risk Calculator, which is based on clinical parameters (AUC 0,66 vs. 054 vs. 0,47, respectively). Moreover, this panel of urinary sEV miRNA is available as a commercial, IVD-certified test—ExoDx Prostate IntelliScore (EPI). Kretschmer et al. (2022) analyzed the performance of the same test. In their study, a comparison of EPI score with post radical prostatectomy histopathological results showed that EPI outperformed PSA, PCPT, and ERSPC in the risk prediction of low-grade cancer. However, as both of the studies’ sEV analysis was performed by commercial laboratories, no technical details of controls were shown.

However, other sEVs carrying non-coding RNAs were also evaluated for their potential diagnostic application. The analysis of urinary sEVs by Wani et al. (2017) revealed that exosomal miR-2909 is a promising biomarker of PCa and a predictor of its aggressiveness. Their research showed better prediction values than other previously evaluated urinary sEV miR-615-3p and serum PSA level. However, this study was conducted on a small group of patients and required confirmation on the bigger group. Another urinary sEV miRNA investigated by Hasegawa et al. (2018) as a potential PCa biomarker was miR-888. Their work revealed a rising level of miR-888 in sEVs from urine after digital rectal examination (DRE) from clinical patients with high-grade PCa and the involvement of the entire miR-888 cluster in PCa cell proliferation, migration, and invasiveness. Moreover, they observed that miR-888 and miR-891a promoted tumor formation in mice, which indicates that these miRNAs are potential therapeutic targets for PCa. Analysis of urinary sEVs by Rodriguez et al. (2017) indicated downregulation of miR-196a-5p and miR-501-3p in PCa samples but needs confirmation on large patient cohorts. The variety in PCa-associated miRNAs between sEVs and other components of urine was presented by Foj et al. (2017). Upregulation of miR-21 and miR-375 found in urinary pellets was also detected in urinary sEVs. However, upregulation of miR-141 found in urinary pellets was not detected in urinary sEVs of PCa patients. The opposite situation was observed for the change of let-7c, in which different levels were observed in sEVs of PCa patients but not in their urinary pellets. An elevated level of miR-21 level in urinary sEVs of PCa patients was also observed by another group, strengthening possible application for PCa diagnosis (Danarto et al., 2020). Lee et al. (2018), in their work, proposed the detection of urinary sEV miR-375 and miR-574-3p using molecular beacons, nano-sized oligonucleotides probes with an internally quenched fluorophore, as a potentially cost-effective and non-invasive tool for PCa detection. One of the biggest advantages of their approach is that this assay is not affected by other urine components that may interfere with the detection of miRNA in sEVs, thus the sample can be applied directly. Li et al. proposed another potential application of urinary sEV miR-375 in PCa diagnosis. The proposed panel of miR-375, miR-451a, miR-486-3p, and miR-486-5p has shown a good 91% sensitivity with high 89% specificity for PCa detection. Moreover, the results for comparison between metastatic PCa and localized PCa have shown that miR-375 can be used to distinguish between these two types of cancer (Li Z. et al., 2021). Urinary sEVs are not the only source of miRNA for potential PCa diagnostic biomarkers. Analysis of miR-1246 in serum sEVs conducted by Bhagirath et al. (2018) showed its superiority to the PSA diagnostic value with higher sensitivity and accuracy. (Malla et al. (2018)performed an analysis of sEVs purified from serum samples of PCa patients undergoing radiotherapy. They observed a change in the let-7a-5p level in high-risk patients before and after radiation and miR-21-5p between intermediate and high-risk patients, suggesting their different roles in both groups. Analysis of sEVs performed by Wang et al. shows the potential of focusing detection of non-coding RNA cargo of sEVs selected by the presence of specific proteins on their surface. In their study, the team showed that the approach of immunomagnetic separation for PSMA or EGFR from pre-purified plasma sEVs resulted in significantly different results compared to the analysis of the total sEV content. The analysis of SAP30L-AS1 and SChLAP1 in sEVs obtained with the method mentioned earlier showed their diagnostic potential for differentiation of BPH and PCa patients’ samples from the healthy group. Moreover, the combination of detection of levels of these two lncRNAs with PSA-level analysis provides a sensitivity of 82.8% with a specificity of 99%. Within the group of PSA levels between 4 and 10 ng/ml, SChLAP1 allowed for differentiation between BPH and PCa with AUC = 0.989 (Wang Y.-H. et al., 2018). Not only the presence of PCa can be detected, but also the state in which the cancer cells are, with analysis of sEV miRNAs. Panigrahi et al. (2018) identified unique miRNAs with differential expression in sEVs secreted from hypoxic PCa cells and suggested their potential usefulness as a hypoxia biomarker in PCa patients. Moreover, they found that sEVs secreted by human PCa cells under hypoxia promote invasiveness and stemness in naive PCa cells. The least examined source of sEVs for PCa diagnosis was semen. Although the study of Barcelo et al. (2019)showed that it should not be neglected. They found that combining PSA levels with three sEV miRNAs (miR-142-3p, miR-142-5p, and miR-223–3) significantly raised discrimination of PCa from benign prostate hyperplasia (BPH), which is the most challenging to avoid overtreatment of non-malignant changes. A more complex sEVs carried miRNA analysis from both semen, and post-DRE urine was performed by Ruiz-Plazas et al. (2021). In their study, researchers compared the tumor necrosis factor–like weak inducer of apoptosis (TWEAK) associated miRNAs and found elevated levels of miR-221-3p, -222-3p, and -31-5p from semen of high-risk PCa patients compared to the low-risk group. In the case of post-DRE urine, lower levels of miR-193-3p and -423–5 were noted in the high-risk group compared to the low-risk peers. The proposed panel of semen sEVs miR-221-3p, miR-222-3p, and TWEAK showed 85.7% specificity and 76.9% sensitivity for aggressive PCa classification. Albino et al. (2021)have found that in serum sEVs of patients with primary or metastatic PCa patients, miR-424 was present, while it was not detectable in patients with BPH. Moreover, *in vitro* and *in vivo* analyses of sEVs loaded with miR-424 have shown that their uptake by PCa cells was causing greater ability of these cells to promote the tumor-sphere formation and has enhanced their tumorigenic potential, making it not only diagnostic but also treatment target. Such dual potential properties were also found for miR-217 and miR-23b-3p by Zhou et al. (2020) In their study, analysis of plasma sEV miRNA has shown that deregulation of these two molecules was present not only in PCa patients samples but also in PCa cell line models. Analysis of the animal model revealed that sEVs carrying higher levels of these miRNAs caused formation of tumors with greater mass and higher vimentin expression. These results show potential involvement of cancer sEVs in acquiring invasiveness by PCa cells and contribution to distant metastasis formation.

#### 3.1.2 Circular RNA (circRNA)

Circular RNA (circRNA) is another non-coding type of RNA that is believed to be associated with tumorigenesis and metastasis and can be carried by sEVs. Analysis of circRNA of blood sEVs in PCa patients showed that circ_0044516 was significantly upregulated compared to healthy control. Moreover, further analysis showed that the downregulation of circ_0044516 inhibited the proliferation and migration of cancer cells *in vitro,* revealing its therapeutic target potential (Li et al., 2020).

#### 3.1.3 mRNA

Messenger RNAs (mRNA) carried by sEVs were also checked for their diagnostic and prognosis value in PCa. Joncas et al. (2019) analyzed androgen receptor splice variant 7 (AR-V7) mRNA levels in PCa patients with different stages of the disease. Their findings showed that sEV AR-V7 level correlated with poorer prognosis in castrate-resistant prostate cancer (CRPC) patients. However, another study by Nimir et al. (2019) revealed that for this clinically significant PCa biomarker analysis, circulating tumor cells (another approach to the liquid biopsy method) should be chosen over sEVs. Therapy management and prognostic potential of plasma sEVs carrying mRNA in CRPC was also shown by Zhu et al. The results of the analysis of sEV TUBB3 mRNA levels showed dependence between the elevated TUBB3 level and poor PSA progression-free time. However, the lack of a control group, small group of patients, and lack of in-depth analysis limit the complete understanding of the underlying mechanism (Zhu et al., 2021). The panel of mRNAs carried by serum sEVs for detection of PCa was proposed by Ji et al. (2021). With the results of their study, a panel of sEVs carrying CDC42, IL32, MAX, NCF2, PDGFA, and SRSF2 mRNAs showed promising performance in predicting biopsy results as well as distinguishing PCa patients from a healthy control. However, validation with a more extensive study group is needed to confirm obtained diagnostic performance.

#### 3.1.4 Proteins

RNAs are not the only cargo of sEVs analyzed as a potential PCa biomarker. Another interest group is proteins carried by sEVs and present on their surface. A study by Logozzi et al. (2017) revealed that PCa patients’ plasma contained four-fold more sEVs presenting PSA than the healthy control. Moreover, in their subsequent study on a larger group, researchers provided data indicating that plasmatic CD81 ^+^ sEV PSA level analysis, with adjusted cut-off, provided 100% sensitivity and specificity in distinguishing PCa patients from the healthy control group. Moreover, 98% of sensitivity and 80% of specificity discriminate BPH patients from healthy control and PCa patients (Logozzi et al., 2019). Another study of this group showed that plasmatic sEVs from PCa patients carried an increased amount of carbonic anhydrase IX (CA IX). This finding was also supported by an analysis of the intraluminal pH of sEVs, which can affect the activity of CA IX. Their results showed, for the first time, acidic pH of PCa patients’ sEVs, which can further help to better understand the sEV influence on the microenvironment of prostate tumors (Logozzi et al., 2020). A study of Li et al. showed the potential of sEV proteins in overcoming problems with distinguishing BPH and PCa. They found that sEV ephrinA2 was superior to serum PSA in distinguishing PCa patients from BPH. Their results also showed the necessity of separate analysis of free proteins and proteins being part sEVs, as the diagnostic efficiency of sEV ephrinA2 was superior to that of whole serum ephrinA2. Moreover, they found that the sEV ephrinA2 expression was positively correlated with TNM staging and Gleason score of PCa patients, which would allow better treatment decisions without invasive procedures (Li et al., 2018). Another potential sEV protein to distinguish between BPH and PCa was found by Panigrahi et al. (2019). By mass spectrometry–based proteomic analysis of serum sEVs, they found a decrease in filamin A expression in PCa patients compared to BPH. However, they also pointed necessity of distinctive analysis for different ethnic groups, as they found protein isoform 2 of filamin A higher loading (2.6-fold) in sEVs from African Americans with PCa, but a lesser loading (0.6-fold) in sEVs from Caucasian men with PCa, compared to race-matched healthy individuals. The dual potential of sEVs in PCa, as diagnostic and therapeutic targets, was also presented by Krishn et al. (2019). They showed that sEV αvβ3 integrin in PCa patients could be a clinically valuable non-invasive biomarker. Moreover, αvβ3 integrin is suspected to be involved in the pathogenesis o PCa, thus sEVs carrying αvβ3 integrin could also be a therapeutic target. Not only the presence but also the activity of proteins carried by sEVs was analyzed for potential PCa diagnostic significance. In a study by Kawakami et al. (2017), higher activity of serum sEV GGT1 enzyme was noted in PCa patients compared to BPH patients, providing a potential stratification marker.

#### 3.1.5 Lipids

Another group of sEV cargo analyzed as potential PCa biomarkers is of lipids. . Skotland et al. (2017)performed lipidomic analyses of urinary sEVs and discovered alterations in nine lipid species levels between PCa patients and healthy control group samples. The most significant differences were observed for phosphatidylserine and lactosylceramide. Moreover, alterations in specific sphingolipid lipid classes were observed. Another lipidomic analysis of urinary sEVs, separated by flow field-flow fractionation, was performed by Yang et al. (2017). In their study, researchers found with nUPLC-ESI-MS/M analysis that PCa patients’ urinary sEVs had higher 22:6/22:6-phosphatidylglycerol and TAG levels, with lower levels of (16:0.16:0) and (16:1, 18:1)-DAG species, compared to healthy peers. Their results showed potential for utilization of this combination of methods to allow for fast sEV analysis. However, high technical requirements and small study groups limit their results’ significance.

#### 3.1.6 Metabolites

Another distinct method of sEV analysis for PCa diagnosis is metabolomics analysis. Clos-Garcia et al. (2018) observed alterations in phosphatidylcholines, acyl carnitines, citrate, and kynurenine in urinary sEVs of PCa patients. Moreover, they found an increased level of dehydroepiandrosterone sulfate, suggesting an elevation of androgen synthesis typical for PCa and its potential prognostic value. Distinct metabolomics analysis of urinary sEVs performed by Puhka et al. (2017) indicated decreased levels of glucuronate, D-ribose 5-phosphate, and isobutyryl-L-carnitine in pre-prostatectomy samples compared to the healthy control and post-prostatectomy samples. However, changes were only detected from sEVs by normalization to EV-derived factors or metabolite ratios, not from the original urine samples. With the knowledge about the specific enrichment of metabolites and normalization methods, EV metabolomics could be used to gain novel biomarker data not revealed by the analysis of the original EV source materials.

### 3.2 Urinary bladder cancer

Bladder cancer (BCa) is the second most common type of UTC, thus, there are also many studies analyzing the potential use of sEVs for diagnosis of tumor formation, but also stratification of aggressiveness and muscle invasiveness.

#### 3.2.1 lncRNA

The largest group of recently evaluated particles in BCa-associated sEVs analysis is long non–coding RNAs (lncRNA). A study conducted by Wang et al. evaluated the potential of serum sEV H19 in BCa diagnosis and prognosis. Their results revealed that the concentration of H19 was significantly higher than that in sEV-depleted supernatants in serum, indicating that H19 is distributed mainly as sEV cargo. Analysis of BCa patients’ sera showed that H19 was significantly downregulated in postoperative samples compared to the preoperative samples. Moreover, high H19 expression was connected with significantly poorer prognosis in analyzed patients (Wang J. et al., 2018). Urinary sEVs carrying lncRNAs were also evaluated, showing the potential of this population in BCa diagnosis. Chen et al. (2022) analyzed TERC levels in urinary sEVs of BCa patients and compared them to the healthy group. The obtained results indicated promising diagnostic accuracy (sensitivity: 78.86% and specificity: 77.78%), better than FDA-approved NMP-22 Elisa test (60.67% and 74.6%, respectively). Another study evaluating lncRNA potential in BCa diagnosis was conducted by Yazarlou et al. (2018a). In this study, not a single lncRNA but a whole panel was evaluated to identify transitional cell carcinoma. The proposed panel of UCA1-201, UCA1-203, MALAT1, and LINC00355 had 92% sensitivity and 91.7% specificity for diagnosing BCa. Another set of lncRNAs was proposed by Zhang et al. The researchers proposed the use of three lncRNAs from serum sEVs: PCAT-1, UBC1, and SNHG16. Obtained sensitivity and specificity of the proposed markers’ panel (80 and 75%, respectively) in the big patients’ group (*n* = 320) were better in discriminating BCa patients from healthy control than urine cytology. However, considering precipitation as a method of sEV separation from as complex biofluid as serum, a more detailed functional study concerning the method of transport of selected lncRNAs is needed (Zhang S. et al., 2019). A distinct set of sEV lncRNAs, including the aforementioned PCAT-1, in urine samples of the BCa patients with promising diagnostic performance was found by Abbastabar et al. (2020) Their results have shown ANRIL and PCAT-1 potential for low-grade (T1 and T2) detection. The sensitivity and specificity of those markers alone were promising (ANRIL: 46.67 and 87.5%; PCAT-1: 43.33 and 87.5%); however, a study group was relatively small and required confirmation on a larger group. Moreover, combination with other markers, such as in the previous study, might raise the sensitivity, which is relatively lower. Another study using the already mentioned lncRNAs combined in one panel is the study of Zhan et al. (2018). They analyzed a panel of three lncRNAs: MALAT1, PCAT-1, and SPRY4-IT1 from urinary sEVs. Obtained results indicated high sensitivity and specificity of the constructed panel, superior to AUC to urine cytology in BCa detection . Hypoxia in BCa cells can also be detected with sEV analysis. Xue et al. (2017)demonstrated that cells exposed to hypoxic conditions release sEVs containing a higher amount of lncRNA-UCA1 than those under normoxic conditions. Analysis of expression levels of lncRNA-UCA1 in the human serum–derived sEVs of BCa patients revealed its higher levels than healthy controls. Moreover, *in vitro* and *in vivo* experiments, they found that sEVs released by BCa cells exposed to hypoxia promote tumor growth and progression through epithelial-to-mesenchymal transition (EMT). Thus, sEV lncRNA-UCA1 in the human serum has the possible use as a diagnostic biomarker for BCa and therapeutic target. Other sEV lncRNAs that were found to be involved in EMT in BCa are LINC00960 and LINC02470. Huang et al. (2020) have found that BCa cell–derived sEVs from cells with knockdown of either of these lncRNAs have shown a significantly lower effect on viability, migration, invasion, and clonogenicity of recipient cells than wild-type BCa cell sEVs. Moreover, lower EMT-associated molecule expression was lower in cells treated with LINC00960 and LINC02470-depleted sEVs. Other lncRNAs with potential dual-use, both diagnostic and therapeutic, were proposed by Zheng et al. Their study showed that PTENP1 was mainly wrapped by sEVs. Analysis of the sEV PTENP1 level could distinguish patients with BCa from healthy controls. Moreover, sEVs derived from normal cells transferred PTENP1 to BCa cells, reducing the progression of BCa *in vitro* and *in vivo*. Moreover, sEV PTENP1 mediated the expression of PTEN by competitively binding to miRNA-17. The finding suggests that sEV PTENP1 participates in normal-cell-to-bladder-cell communication during the carcinogenesis of BCa, and could be used in its therapy (Zheng et al., 2018). A different approach combining analysis of sEVs carrying lncRNAs and mRNAs was also proposed. In the study of Huang et al. (2021), the panel of two lncRNAs: MIR205HG and GAS5 and three mRNAs: KLHDC7B, CASP14, and PRSS1, based on RNA sequencing of the BCa cells was proposed. The validation of the selected panel on urinary sEVs from BCa patients and healthy controls revealed that the diagnostic accuracy of the panel was better than the analysis of the mRNA/lncRNA molecules alone. The overall panel performance showed sensitivity and specificity of 88.5 and 83.3%, respectively, presenting the potential for mixing distinct classes of molecules into single panels for better diagnostic accuracy.

#### 3.2.2 miRNA and mRNA

miRNAs and mRNAs carried by sEVs were also evaluated as potential markers of BCa. Baumgart et al. (2017) analyzed miRNA levels in urinary sEVs and compared them to levels in corresponding carcinoma tissues. Their results revealed that levels of miRNAs in urinary sEVs of invasive BCa cells differ compared to those of non-invasive BCa. Among the eight suspected sEV miRNAs, on the basis of *in vitro* study, only miR-200a-3p had significantly different levels between invasive and non-invasive BCa patients’ urine samples. This change also corresponds to the deregulation of miR-200a-3p in the biopsy samples. For the rest of the analyzed sEV miRNAs, no significant difference was found between invasive and non-invasive BCa samples. A panel of two miRNAs carried by urinary sEVs was proposed by El-Shal et al. In their study, the diagnostic potential of elevated levels of miR-96-5p and miR-183-5p was shown. Moreover, higher sensitivity and specificity of a combination of these markers were shown than each of them alone, and the potential connection with cytology results was proposed. However, analysis on a larger group of patients is necessary (El-Shal et al., 2021). A more complex analysis of the involvement of miRNAs in BCa was performed by Lin et al. (2021) where the potential of urinary sEV miR-93-5p was analyzed. The comparison of sEVs from BCa patients and healthy counterparts revealed a significantly higher level of miR-93-5p in cancer patients; moreover, correlation with the pT stage was also noticed. The additional animal model study revealed the miR-93-5p-driven promotion of proliferation and migration of BCa cells by targeting BTG2. However, the diagnostic performance needs to be validated on a larger group of patients. Similar to PCa, miRNAs from sEVs possess dual diagnostic and therapeutic properties. An example of such miRNA is miR-633b. In the study of Yin et al. (2020),the significantly elevated level of miR-663b was found in serum sEVs of BCa patients. Further *in vitro* research revealed that miR-633b promotes BCa cell proliferation, plays a role in EMT, and promotes tumor development by targeting the Ets2-repressor factor. Another example of diagnostic/therapeutic target sEV miRNA is miR-4644. The research study of Yan et al. (2020)has shown that miR-4644 is upregulated in plasma sEVs of BCa patients compared to the healthy control. Moreover, *in vitro* results have shown that this miRNA downregulates UbiA prenyltransferase domain–containing protein 1, which is responsible for suppressing BCa growth. Additionally, utilizing a mouse model, the potential of therapeutic inhibition of miR-4644 was presented, as after administration of antagomir-4644, suppression of BCa tumorigenesis was observed. Coding RNAs carried by sEVs were also found to possess diagnostic potential. The analysis of urinary sEVs of a large group of BCa patients revealed significantly higher CA9 levels compared to healthy volunteer samples. However, lack of analysis of in-depth analysis does not disqualify the involvement of other molecules’ transportation pathways, in this phenomenon (Wen et al., 2021). Yazarlou et al. (2018b) analyzed mRNAs of cancer-testis antigens in urinary sEVs and compared their level in patients with BCa, patients with other bladder-associated diseases, patients with BPH, and healthy controls. Their results showed significantly higher levels of MAGE-B4 in BCa compared to healthy control. However, the elevated expression of MAGE-B4 was even higher in BPH patients. A higher level of NMP22 was observed in BCa patients compared to the BPH group. These results show that a proper panel of urinary sEV mRNA can be used in non-invasive BCa diagnosis; however, their potential for clinicopathological discrimination is limited.

#### 3.2.3 Proteins

Proteins from urinary sEVs are also examined for their potential diagnostic value in BCa. Silvers et al. (2017)examined sEVs released from muscle-invasive bladder cancer cell line and normal uroepithelial cell line and performed proteomics analysis. The results indicated six potential proteins associated with inflammation and angiogenesis signaling pathways. Further analysis of urine samples from BCa patients revealed more than fifteen-fold higher levels than healthy control in three investigated proteins: HEXB, S100A4, and SND1. However, the relatively small group investigated in this study causes the necessity of repetition of such analysis in a larger group . A more widespread proteomic analysis of urinary sEVs was performed by Tomiyama et al. (2021). In their study, they have compared proteins from urinary sEVs and tissue-exudative sEVs from BCa patients. With the use of tandem mass tag (TMT)–labeling liquid chromatography (LC–MS/MS) analysis, they have discovered 22 proteins of potential significance, and further evaluated the more extensive group with a targeted approach by SRM/MRM analysis. That analysis revealed significantly different levels of six proteins in urinary sEVs compared to healthy controls: HSP90, SDC1, MARCKS, MARCKSL, TJP2, and CD55, with the diagnostic potential of the first three proteins higher than urine cytology. However, study results require validation on a bigger group of patients, and they provide the basis for using less advanced methods than the ones used to discover these markers.

### 3.3 Renal cell carcinomas

Renal cell carcinomas (RCCs) are the least common group of UTC. However, the potential diagnostic role of sEVs in them was also evaluated in the last years.

#### 3.3.1 miRNA and lncRNA

The most often evaluated group of sEV cargo in this cancer group was miRNA. Kurahashi et al. (2019)performed the analysis of sEVs released from Xp11 translocation RCC. They found elevated levels of miR-204-5p in urinary sEVs from transgenic mice overexpressing the human PRCC-TFE3 fusion gene compared to control. This finding was also confirmed with the same results from urine samples of 20 patients with Xp11tRCC. The most interesting is that an elevated level of sEV miR-204-5p in transgenic mice was found before overt tumor formation, indicating that sEV miR-204-5p has the potential for very early detection of Xp11tRCC at the pretumorigenic stage. Another miRNA from sEVs was evaluated by Fujii et al. (2017). They found that a higher level of miR-224 in serum sEVs of RCC patients was associated with shorter progression-free survival, cancer-specific survival, and overall survival compared with the low-expression group, showing its prognostic potential. In additional *in vitro* analysis, they found that miR-224 has an oncogenic function related to cell-to-cell interaction regarding cancer invasion and metastasis in RCC, and thus it is also a potential therapeutic target. High-throughput sequencing of plasma sEVs’ miRNAs of RCC patients, performed by Xiao et al. (2020) revealed different levels of miR-92a-1-5p, miR-149-3p, and miR-424-3p when compared to the healthy control. The diagnostic potential of every one of these molecules alone, confirmed by qPCR analysis, was very promising (87.5, 75, and 75%: sensitivity and 77.3%, 72.7%, and 81.8%: specificity) with the potential to multiplex it for even better results. One of the problems with ccRCC management is the analysis of tyrosine kinase inhibitor (TKI) resistance, as this subtype of cancer is more susceptible to metastasis. Comparative analysis of sEVs released *in vitro* by TKI-sensitive and TKI-resistant ccRCC cells revealed that miR-549a plays the most important role in the metastatic process among differentially carried miRNAs. sEVs secreted by TKI-resistant cells had a bigger impact on vascular permeability, proven by Western blot analysis of related proteins and analysis with miR-549a mimetic, which confirmed its predominant role in this process. Moreover, sEVs released by these two cell types differed, as TKI-resistant cells secreted smaller sEVs, compared to TKI-sensitive cells. The *in vivo* study confirmed that TKI-resistant ccRCC cell–derived sEVs had a higher metastasis promotion potential, showing the importance of clinical evaluation of this potential prognostic marker (Xuan et al., 2021). Another non-coding RNA carried by sEVs released from ccRCC cells, which was found to be involved in metastasis by rising cell invasion and migration, is MALAT1. A study of Jin et al. (2021) showed that sEVs released by RCC cell line 786-O significantly affected other RCC cell lines raising their aggressiveness. Functional assays revealed that sEVs carrying MALAT1 were responsible for the rise of invasion and migration potential of cells. Moreover, the *in vivo* study involving selective MALAT1 inhibition confirmed *in vitro* results, showing that RCC cell–derived sEVs carrying MALAT1 were promoting transplanted RCC cell growth and metastasis. However, these *in vitro* and *in vivo* studies require confirmation in clinical samples. A similar interplay of RCC cell–derived sEVs promoting a more aggressive phenotype of ccRCC cells was observed by Li et al. They found that RCC cell–derived sEVs contained different levels of carried miR-15a; moreover, silencing or overexpression of this miRNA was changing cell migration and apoptosis, revealing tumor growth modulating potential. The functional analysis revealed a similar effect when cells were treated with miR-15a- containing sEVs. Despite this *in vitro* analysis requiring clinical confirmation, they provide the basis for this potential prognostic marker evaluation (Li D.-Y. et al., 2021). A more specific study was conducted by Zhang et al. (2018) where EpCAM positive sEVs derived from serum were evaluated. Comparing samples from clear-cell RCC (ccRCC) patients before and 7 days after surgical tumor removal revealed sEVs miR-210 and miR-1233 at significantly lower levels postoperatively. This fact indicates that higher levels of miR-210 and miR-1233 in EpCAM positive sEVs have potential as biomarkers for diagnostic and monitoring purposes in ccRCC. The proposition of a different approach for analysis of miRNA levels in sEVs was proposed by Crentsil et al. (2018). They conducted a comparison analysis of sEVs released from the ccRCC cell line and proximal tubule–derived cell line. The modification of analysis they proposed in comparison, not only based on the miRNA level in sEVs but also in originating cells. According to these conditions, they found out that even in a simplified *in vitro* system where miRNA in secreted sEVs might be expected to have a higher correlation with cellular miRNA, only miR-150 and miR-205 were strongly correlated with cellular levels, and of those two, only miR-205 reached significance. On basis of this *in vitro* analysis, downregulation of miR-205 was proposed as a marker of ccRCC, which needs to be examined in ccRCC patients.

#### 3.3.2 Proteins

Analysis of proteins carried by sEVs as RCC biomarkers was also performed. Jingushi et al. (2018)conducted a study analyzing sEVs released directly from tissue in a model designed by them. They compared the protein composition of sEVs released from normal tissue, ccRCC tissue, and ccRCC cell lines. The results of quantitative LC/MS revealed that azurocidin (AZU1) was highly enriched in tumor sEVs. Additional analysis of serum sEVs from ccRCC patients showed similarly higher AZU1 levels, showing its non-invasive diagnostic potential. In many cases in the development of ccRCC abnormalities, an epidermal growth factor receptor (EGFR) is involved. Zhao et al. (2019) analyzed urinary sEVs of ccRCC patients for abnormalities in levels of proteins involved in EGFR signaling pathways. They found that SHC1 was significantly overexpressed in high-grade ccRCC and correlated with poor prognosis. Further analysis revealed that both polymerase I and transcript-release factor (PTFR) expression were regulated by SHC1. The abnormal SHC1-increase PTRF, detected in sEVs from urine, can be a potential marker for ccRCC diagnosis and treatment. Involvement of sEVs in EMT in ccRCC was also analyzed by Wang et al. They showed that among sEVs released by cancer stem cells (CSCs) from ccRCC patients, most of them were CD103 positive. Moreover, CD103 guided CSC sEVs to target cancer cells and organs, conferring the higher metastatic capacity of ccRCC to lungs, suggesting CD103 + sEVs as a potential metastatic diagnostic biomarker (Wang L. et al., 2019).

#### 3.3.3 mRNA

mRNAs carried by sEVs also provide an essential role in RCC diagnosis. A study of Marek-Bukowiec et al. (2021) has shown that RNA sequencing data from sEVs separated from the urine of early-stage RCC patients revealed significantly different signatures compared to healthy volunteers. In their results, levels of five coding RNAs: NME2, AAMP, CAPNS1, VAMP8, and MYL12B were significantly higher and pose a potential for checking a larger group of patients to confirm these preliminary results.

### 3.4 Cancer-cancer microenvironment interplay

Cancer cell–derived sEVs are not the only population that might serve for the UTC diagnostic and prognostic purposes. An interesting group for analysis is cells surrounding the tumor, especially cancer-associated fibroblasts (CAFs) and sEVs secreted by this type of cells. The prognostic significance of CAF-secreted sEVs in prostate cancer was studied by Shan et al. (2020). Their results have shown that miR-432-5p carried by CAFs sEVs promote chemotherapy resistance by targeting the TGF-β pathway. In the *in vitro* setting, they have shown that CAF-derived sEVs were inducing docetaxel, taxane, and bicalutamide resistance in human prostate cancer cell lines. By use of membrane permeable miR-423-5p inhibitor, they have shown that this molecule is responsible for the aforementioned effect, showing not only the potential mechanism of chemoresistance spreading but also prognostic marker significance in choosing treatment options. In bladder cancer, a similar mechanism of drug resistance modulated by CAF-secreted sEVs was observed. A study performed by Shan et al. (2021) revealed that CAF-derived sEVs promoted metastasis, EMT, and doxorubicin and paclitaxel resistance in the *in vitro* model. However, in this case, the functional analysis revealed the involvement of sEV miR-148b-3p, affecting PTEN in sEV- internalized cancer cells as the mechanism. These results show that in different types of cancer, different sEVs carried molecules that are involved in similar cancer progression and chemoresistance mechanisms. Involvement of CAF-derived sEVs in ccRCC progression was also examined in recent studies. The *in vitro* study by Liu et al. (2021) has shown that sEVs released from ccRCC patients’ CAFs carrying miR-224-5p were promoting more malignant behavior of ccRCC cells, rising cell proliferation, migration, and invasion. Thus, this sEV-carried miRNA should be explored as a potential therapeutic target and as a prognostic marker in ccRCC. CAFs are not the only type of cells with cancer-modulating properties. Hypoxic tumor–associated macrophages released the sEV effect on RCC cells was also evaluated. qPCR analysis of sEVs released by macrophages in normoxia and hypoxia revealed higher levels of miR-155-5p in sEVs under hypoxic conditions. Moreover, higher levels of this miRNA were noticed in ccRCC cells when co-cultured with hypoxic tumor–associated macrophages, as well as dependence of blockade of internalization of sEVs to RCC cells. The retrospective analysis of the RCC patient TCGA database revealed a poor prognosis for high miR-155-5p patients, showing the potential of this sEV-carried molecule as a prognostic marker (Gu et al., 2021).

## 4 Challenges of sEVs as biomarkers for UTC

### 4.1 Extracellular vesicle separation and analysis challenges

Despite the rising number of EV-focused studies, the most significant limitation in EV studies is establishing and sticking to the standardized methodology approaches (Gandham et al., 2020). Because of the nano-size nature of EVs, separation and analysis methods need to be adequately controlled to avoid misinterpretation and distinguish the adequately obtained data from impurities or artifacts (Soekmadji et al., 2020). Currently utilized methods for EV purification are imperfect and present the consensus between yield and purity of the obtained EVs, and ones’ choice needs to be guided by the study’s primary aim. Only once separated EVs have been appropriately characterized by analysis of size, number, and positive and negative markers, a downstream analysis should be considered (Thery et al., 2018). Moreover, evidence of two different classes of functional extracellular nanoparticles, supermeres and exomeres, carrying biologically relevant cargo, possessing size within the lower range of size of sEVs, raises even more challenges (Zhang et al., 2021).

When discussing sEV purification methods, several factors need to be discussed as they will affect obtained results in downstream analysis: yield, purity, complexity, and cost of the method. As every method is based on a different principle and sEV properties, the obtained EVs may differ, not only in terms of presence and levels of impurities. The ultracentrifugation method is one of the most commonly used and mature methods and is considered a “Gold standard.” The main principle of this method is differences in the density and size of constituents of the sample. This method provides concentrated sEVs in the form of a pellet or a phase when the differential gradient is used. This method is relatively cheap when not incorporating the price of the ultracentrifuge. However, it is time-consuming, the reproducibility is poor, and it is operator dependent. Moreover, contamination from protein aggregates and small organelles is possible (Tauro et al., 2012). The second most common method used is polymer precipitation. This method is based on using a polymer solution to lower EV solubility, requiring lower centrifugation speeds to obtain separated EVs. Even though the complexity and price of this method are relatively low, this method is associated with a high level of protein impurities. As it is not specific for EVs only, it should be avoided unless an additional purification step is performed, especially when downstream analysis of proteins carried by EVs is considered (Ryu et al., 2020). Another common method used for separating EVs is membrane affinity–based techniques, mainly in the form of spin columns. This method utilizes the fact that phosphate groups of phospholipids negatively charge the surface of the exosomal membrane. Thus, using metal oxides which bind with phosphate groups, relatively quick and easy methods were proposed. However, this method does not provide any EV class–specific properties, a proper pre-purification is necessary if a specific class of EVs is the subject of the study (Stranska et al., 2018). Another type of EV purification method arising is size exclusion chromatography (SEC). This method utilizes a differential elution profile of particles of different sizes while running through the stationary phase. The stationary phases can be modified not only by pore size but also the polymer type, allowing exclusion of protein aggregates. However, relatively high purity of samples obtained with this method, contamination from lipoproteins such as chylomicrons and VLDL, especially while small-sized EVs are a target, is undeniable. The rising popularity of this method is also leading to the creation of less user-dependent automated systems (Monguio-Tortajada et al., 2019). Another popular EV separation method is immune affinity–based techniques. This method is based on capturing EVs with specific proteins on their surface, most commonly the tetraspanins. This expensive method provides high purity but low yield EVs with a relatively straightforward procedure. Another disadvantage is the fact that EVs released by some of the cells, for example RBCs, do not present characteristic tetraspanins on their surface; thus, this method may result in omitting some of the potential essential populations of EVs if used as a primary separation technique (Kuo et al., 2017). However, combining this technique as a secondary enrichment for cell-specific markers may provide a more specific approach to the analysis (Mathivanan et al., 2010).

Another critical parts of EV analysis are size and concentration analysis methods. As this might also impact the proper analysis of obtained data, it should not be omitted during the control of obtained samples. Among the most common techniques, two optic-based methods: dynamic light scattering (DLS) and nanoparticle tracking analysis (NTA), take the lead. However, both of these techniques, especially DLS, rely on averaging, thus, the sampling might bias their results, and their accuracy in lower detection limit might also be compromised. New techniques such as tunable resistive pulse sensing (TRPS), allowing analysis at a single particle level, are proposed to overcome these limitations. However, time required for multiple samples as well as the small size of the analysis range within a single analysis of TRPS provides the place for improvement for working, especially with more heterogenous samples. One of the most significant limitations of the studies analyzed in this study is the lack of analysis of negative/impurities markers, especially in more complex samples such as plasma or serum, where different components might be responsible for the obtained results.

Another essential factor is the gathering of the material for sEV analysis. As presented in the study of Hiltbrunner et al. (2020), comparative proteomic analysis of sEVs from urine collected directly from the bladder and ureter revealed differences even in the size distribution of obtained sEVs. Moreover, upregulation of several proteins from bladder samples compared to ureter samples was noted, showing the influence of the site of urine gathering).

Unifying reporting of the EV analysis results is also very important, resulting in rising reproducibility and significance of the obtained data. This reporting unification applies to the method of EV separation and downstream analysis and the gathering and handling of the matrix used as the source of EVs (Van Deun et al., 2017).

### 4.2 Detection strategies

Most of the methods used for downstream analysis of sEV cargo do not differ from methods used in more classic cellular analysis. The biggest challenge is facilitating methods with high sensitivity because of the relatively low amount of molecules carried by sEVs. Another challenge is setting appropriate biological and technical controls not to be misled by artifacts or co-separated molecules not carried by sEVs and calibrators for instrument set-up.

In studies analyzing the RNA content of sEVs, qPCR takes the lead, as it is the most standardized, proven technique. This approach possesses the potential for analysis of a high number of samples within a short time period. However, one of the pitfalls is necessity of control of sample loading, in contrary to cells, where reference genes controls are used. For that reason, spike-in controls can be used; however, there are not many studies that use this type of control. In many cases, qPCR is preceded by RNA sequencing as a more widespread, preliminary analysis before using more focused qPCR. On the other hand, different approaches are also made, for example, with molecular beacon (MB) technologies. This approach might provide more complex results, as with proper modification of beacons, no lysis of EVs is required, and multiplexing with nano-flow cytometry or super-resolution microscopy for simultaneous detection and colocalization of other types of molecules is possible (de Oliveira et al., 2020). An even more complex analysis of simultaneous *in situ* sEV miRNA and surface protein content detection for PCa diagnosis was also proposed by Cho et al. (2019). In their study, combination of immunocapturing of the vesicles with magnetic beads, RNA detection with MB, and detection of surface proteins with antibodies was proposed and its performance was proven with the *in vitro* PCa cell–derived sEV model.

In the case of protein detection, the most commonly used technique is ELISA. However, as many points of care have ELISA assay readers, relative ease, and potential for implementation in routine diagnostic procedures, much work is required for proper standardization of this technique. The second most common technique is Western blotting, as the technique provides the possibility for a broad analysis of isolated proteins. The main challenge of sEV analysis result interpretation is the way of normalization of data, as not many studies report it because normalization for the total protein content versus per vesicle content or per starting material volume might provide different conclusions. Another limitation is the time consumption of the technique and low theoretical throughput for the clinically relevant application. The third most commonly used protein analysis method is mass spectrometry, which requires very expensive equipment and experienced personnel for proper result analysis. This approach is most commonly used for broad proteomic analysis as a preliminary search of proteins of interest, which are furtherly analyzed with less sophisticated methods. Finally, the least utilized method is nano-flow cytometry–based assays. One of the most probable reasons is that this type of test requires more validation before widespread use, as currently used machines pose a limit of size detection of analyzed particles, higher than lower size range of sEVs, which does not allow proper analysis of the total sEV population.

**FIGURE 4:**
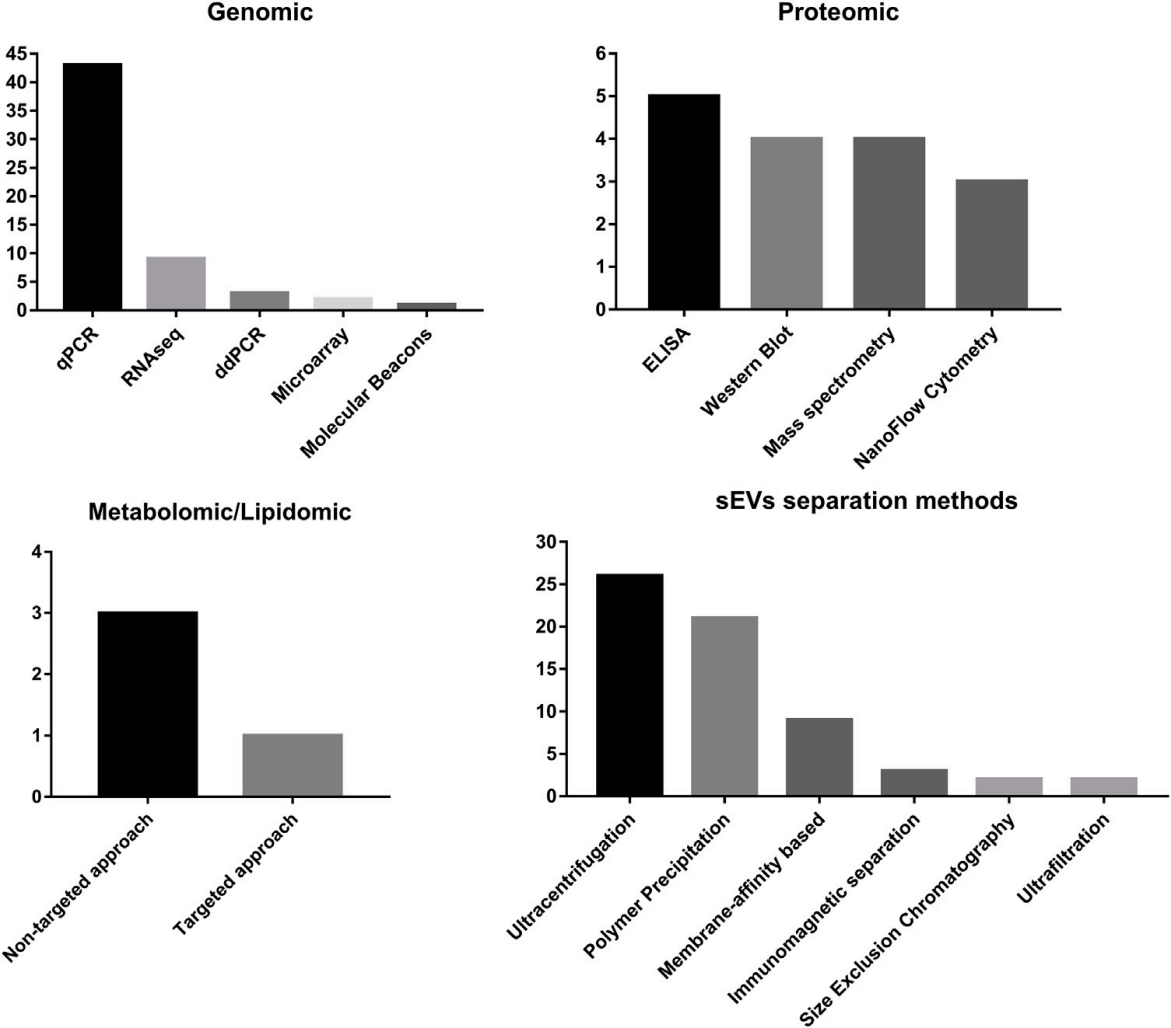
Summary of frequency of methods used for sEV separation and methods used for detection of different classes of molecules carried by sEVs.

In the case of metabolomic and lipidomic analyses, mass spectrometry is the only method used, and in most cases, non-targeted analyses are performed over the targeted approach (Figure 4).

However, in most cases, more in-depth analyses of the involvement of discovered biomarkers in the carcinogenesis process, with the gain of function/loss of function analysis, are only performed when the therapeutic possibility of biomarker use is within the scope of the studies. This fact limits proper understanding of the significance of analyzed molecules. A similar limitation applies to the proper analysis of the specificity of sEV-based transport of analyzed molecules.

Not only are sEV-associated biomarkers evaluated, but efforts to make diagnosis procedures with the use of sEVs easier are made, allowing more widespread use of sEVs in UTC diagnosis. One of such approaches was proposed by Li et al. The platform they proposed is composed of superparamagnetic conjunctions and molecular beacons (SMC-MB). This method is based on aptamer immunoaffinity with ultra-sensitive detection efficiency and reversible isolation capacity. Moreover, the additional use of prostate-specific membrane antigen (PSMA) aptamer to detect and capture PSMA-positive sEVs from urine samples provided good diagnostic efficiency for PCa (Li P. et al., 2019). Another distinct approach for sEVs carried miRNA was proposed by Wang et al. (2021). In their study, a biosensor based on magnetic nanoparticles for immobilization of sEV-isolated miRNA and detection with toehold-aided cyclic amplification combined with horseradish peroxidase catalysis. The proposed method resulted in miRNA limit of detection within 10 fM range, and application with plasma sEVs.

### 4.3 Specificity of explored biomarkers

What should be addressed is the fact that some of these sEV markers found to be relevant in UTC are also significant in other types of cancers. This is especially pronounced when molecules involved in the regulation of EMT are discussed. mir200c is such an example, as this miRNA is one of the molecules responsible for EMT inhibition mainly by controlling the ZEB1/2E-cadherin axis. This miRNA additionally control cells’ migration and invasion potential by targeting cytoskeleton regulatory proteins FHOD1 and PPM1F expression in breast and colorectal cancers (Jurmeister et al., 2012; Jiang et al., 2020). The following example of miRNAs of multiple cancer detection potentials is miR-1246. This molecule was found to be promoting tumor angiogenesis, growth, and metastasis in lung, liver, and pancreatic cancers (Chai et al., 2016; Kim et al., 2016; Xu et al., 2017). Moreover, functional studies have shown that exosomal miR-1246 is generated in the non-canonical miRNA biogenesis process, and its packaging into sEVs is highly enriched in cancer cells, showing potential for analysis in other types of cancers (Xu et al., 2019). A different type of multi-cancer purposed sEV marker is EGFR, which in addition to its importance in UTC has been shown to play an important role in gastric cancer to liver metastasis by creating the microenviromental niche (Zhang et al., 2017). Additionally, in non–small–cell lung cancer, it was shown that sEVs carrying EGFR influence resistance to osimertinib treatment (Wu et al., 2021). Another example of a beyond UTC diagnosis sEV marker is S100A4, which in addition to BCa, was also found to be involved in the promotion of metastasis in hepatocellular cancer (Sun et al., 2021).

## 5 Conclusion

In recent years, interest in extracellular vesicles has been continuously rising. An increase in the quality and quantity of performed studies is undeniable. The analysis of potential diagnostic targets of UTC in sEVs is no exception in this case. The most interest is focused on prostate cancer, reflecting both incidence and problems with proper diagnosis compared to bladder and renal cancer. The cargos of sEVs that are mainly evaluated are miRNAs and proteins. However, the rise of interest combined with more complex analyses such as metabolomics and protein modification pattern analysis is also observed. However, considering the recently emerging evidence indicating the involvement of micropeptides encoded by lncRNAs in the cancer development process, the rise in the analysis of this class of molecules might significantly increase (Ye et al., 2020). Nevertheless, recent studies show that not only level miRNA should be considered, but also analysis of their post-transcriptional modifications. It has been shown that methylation of miRNA may compromise the affinity to targeted sequencing inhibiting their regulatory properties, which also might be a prognostic factor in cancers (Cheray et al., 2020). The most common sources of sEVs are urine, as it has direct contact with cancer cells and serum/plasma. However, some studies show that other sources such as semen should not be neglected (Tables 1–3). Regarding the most common methods for sEV separation, ultracentrifugation and polymer precipitation are taking the lead, reflecting the maintained global trends in EV analysis protocols (Gardiner et al., 2016). Considering the aforementioned limitations of these techniques, especially potentially high-protein contaminations, the results might require better validation, to diminish the possibility of influence of co-purified RNA-binding proteins or soluble proteins. As sEVs are very challenging technically and methodologically material, because of their nano-scale size, the proper choice of method of separation and analysis can lead to different results. In particular, results of more sophisticated methods such as metabolomics analysis or analysis of protein modification patterns are prone to change with different methodologies (Freitas et al., 2019; Dudzik et al., 2021).

**TABLE 1:** Summary of the recently evaluated small extracellular vesicle cargo as potential biomarkers for prostate carcinomas. sEVs–small extracellular vesicles, miRNA–microRNA, mRNA–messenger RNA, circRNA–circular RNA, lncRNA–long non–coding RNA.

Cargo	Source of sEV	Example (sensitivity/specificity if available)		Purification method	Reference
miRNA and lncRNA	Blood	miR-1246 (75%/100%)	(75%/100%)	Polymer precipitation	Bhagirath et al. (2018)
		miR-424		Polymer precipitation	Albino et al. (2021)
		miR-217		Ultracentrifugation	Zhou et al. (2020)
		miR-23b-3p			
		miR-21-5p		Polymer precipitation	Malla et al. (2018)
		let-7a-5p			
		SAP30L-AS1	(+PSA = 82.8%/99%)	Polymer precipitation + immunomagnetic separation	Wang et al. (2018b)
		SChLAP1			
	Urine	miR-2909		Polymer precipitation	Wani et al. (2017)
		miR-615-3p			
		miR-888		Ultracentrifugation	Hasegawa et al. (2018)
		miR-196a-5p	(100%/89%)	Sequential centrifugation	Rodriguez et al. (2017)
		miR-501-3p		Ultracentrifugation/polymer precipitation	Foj et al. (2017)/Danarto et al. (2020)
		miR-21			
		miR-200c		Polymer precipitation	
		miR-375			Li et al. (2021b)
		miR-451a			
		miR-486-3p	(91%/89%)		
		miR-486-5p		Polymer precipitation	Lee et al. (2018)
		miR-574-3p		Ultrafiltration	McKiernan et al. (2020)
		PCA3			Kretschmer et al. (2022)
		ERG		
		SPDEF		
	Semen	miR-142-3p		Ultracentrifugation	Barcelo et al. (2019)
		miR-142-5p	(+PSA = 91.7%/43.3%)		
		miR-223–3			
		miR-342-3p	(+PSA = 81.8%/95%)		
		miR-374b-5p
		miR-31-5p		Membrane affinity–based	Ruiz-Plazas et al. (2021)
		miR-221-3p	(+TWEAK = 86%/77%)
		miR-222-3p
circRNA	Blood	circ_0044516		Membrane affinity–based	Li et al. (2020)
mRNA	Blood	AR-V7		Ultracentrifugation/polymer precipitation	Joncas et al. (2019)/Nimir et al. (2019)
				Membrane affinity–based	Ji et al. (2021)
		CDC42			
		IL32			
		MAX			
		NCF2			
		PDGFA			
		SRSF2		Membrane affinity–based	Zhu et al. (2021)
		TUBB3			
Proteins	Blood	PSA (100%/100%)		Ultracentrifugation	Logozzi et al. (2017)/Logozzi et al. (2019)
		Carbonic anhydrase IX		Ultracentrifugation	Logozzi et al. (2020)
		EphrinA2 (88%/80.95%)		Ultracentrifugation	Li et al. (2018)
		Filamin A		Ultracentrifugation	Panigrahi et al. (2019)
		αvβ3 Integrin		Ultracentrifugation	Krishn et al. (2019)
		GGTA1		Ultracentrifugation	Kawakami et al. (2017)
Lipids	Urine	Phosphatidylserine (93%/100%)		Ultracentrifugation	Skotland et al. (2017)
		Lactosylceramide			
		Sphingolipids			
		22:6/22:6-phosphatidylglycerol		AF4	Yang et al. (2017)
		TAG			
		(16:0.16:0) and (16:1, 18:1)-DAG			
Metabolites	Urine	Phosphatidylcholine acyl		Ultracentrifugation	Clos-Garcia et al. (2018)
		Carnitines			
		Citrate			
		Kynurenine			
		Dehydroepiandrosterone sulfate			
		Glucuronate		Ultracentrifugation	Puhka et al. (2017)
		D-ribose 5-phosphate isobutyryl-L-carnitine			

**TABLE 2:** Summary of the recently evaluated small extracellular vesicle cargo as potential biomarkers for urinary bladder carcinomas. sEVs–small extracellular vesicles, miRNA–microRNA, mRNA–messenger RNA, circRNA–circular RNA, lncRNA–long non–coding RNA, SEC–size exclusion chromatography.

Cargo	Source of sEV	Examples (sensitivity/specificity if available)		Purification method	Reference
lncRNA	Blood	H19 (74.07%/78.08%)		Polymer precipitation	Wang et al. (2018a)
		UCA1-201		Membrane affinity–based
		UCA1-203	(92%/91.7%)		Yazarlou et al. (2018a)
		MALAT1		
		LINC00355	
		lncRNA-UCA1 (80%/83.33%)		Polymer Precipitation	Xue et al. (2017)
		PTENP1 (65.4%/84.2%)		Polymer Precipitation	Zheng et al. (2018)
		PCAT-1		Polymer precipitation	Zhang et al. (2019a)
		UBC1	(80%/75%)		
		SNHG16			
	Urine	ANRIL (46.67%/87.5%)		Membrane affinity–based	Abbastabar et al. (2020)
		PCAT-1 (43.33%/87.5%)			
		MIR205HG		Membrane affinity–based	Huang et al. (2021)
		GAS5			
		TERC (78.65%/77.78%)		Polymer precipitation	Chen et al. (2022)
		MALAT1		Membrane affinity–based	Zhan et al. (2018)
		PCAT-1	(62.5%/85%)		
		SPRY4-IT1			
	Conditioned medium	LINC00960		Ultracentrifugation	Huang et al. (2020)
		LINC02470			
miRNA	Urine	miR-200a-3p		Polymer precipitation	Baumgart et al. (2017)
		miR-93-5p (74.1%/90.2%)		Ultracentrifugation	Lin et al. (2021)
		miR-96- 5p		Membrane affinity–based	El-Shal et al. (2021)
		miR-183-5p	(88.2%/87.8%)		
	Blood	miR-633b		Polymer precipitation	Yin et al. (2020)
		miR-4664		Ultracentrifugation + SEC	Yan et al. (2020)
mRNA	Urine	MAGE-B4 (71.7%/66.7%)		Membrane affinity–based	Yazarlou et al. (2018b)
		NMP22			
		CA9 (85.18%/83.15%)		Polymer precipitation	Wen et al. (2021)
		KLHDC7B		Membrane affinity-based	Huang et al. (2021)
		CASP14			
		PRSS1			
Proteins	Urine	HEXB		Ultracentrifugation	Silvers et al. (2017)
		S100A4			
		SND1			
		HSP90 (82.5%/70%)		Ultracentrifugation	Tomiyama et al. (2021)
		SDC1 (82.5%/63.3%)			
		MARCKS (65%/80%)			
		MARCKSL			
		TJP2			
		CD55			

**TABLE 3:** Summary of the recently evaluated small extracellular vesicle cargo as potential biomarkers for renal cell carcinoma. sEVs–small extracellular vesicles, miRNA–microRNA, mRNA–messenger RNA, circRNA–circular RNA, lncRNA–long non–coding RNA, SEC–size exclusion chromatography.

Cargo	Source of sEVs	Example (sensitivity/specificity if available)	Purification method	Reference
miRNA and lncRNA	Urine	miR-204-5p	Immunomagnetic separation	Kurahashi et al. (2019)
	Blood	miR-224	Polymer precipitation	Fujii et al. (2017)
		miR-92a-1-5p (87.5%/77.3%)	Ultrafiltration	Xiao et al. (2020)
		miR-149-3p (75%/72.7%)		
		miR-424-3p (75%/81.8%)		
		miR-210 (70%/62.2%)	Polymer precipitation + immunomagnetic separation	Zhang et al. (2018)
		miR-1233 (81%/76%)		
	Cell conditioned medium	miR-205	Ultracentrifugation	Crentsil et al. (2018)
		miR-549a	Ultracentrifugation	Xuan et al. (2021)
		MALAT1	Ultracentrifugation +	Jin et al. (2021)
			Polymer precipitation	
		miR-15a	Ultracentrifugation	Li et al. (2021a)
Proteins	Tissue	AZU1	Unique protocol + Ultracentrifuation	Jingushi et al. (2018)
	Blood	AZU1 (52.6%/100%)	SEC	Jingushi et al. (2018)
	Urine	EGFR	Ultracentrifugation	Zhao et al. (2019)
		SHC1		
		PTFR		
		CD103	Ultracentrifugation	Wang et al. (2019a)
mRNA	Urine	NME2	Polymer Precipitation	Marek-Bukowiec et al. (2021)
		AAMP		
		CAPNS1		
		VAMP8		
		MYL12B		

The second and significant branch of research about sEVs is finding possible treatment applications of sEVs in cancer treatment. Research teams took several pathways. One of them is using sEVs as a target of the therapy. This approach is strictly connected with hypothesized mechanisms of sEV involvement in metastasis. Many potential targets in this branch are discovered when finding diagnostic biomarkers, such as the ones previously mentioned in this study (Table 4). Another branch, in its foundations, uses the exact mechanism of relatively easy integration of sEVs with recipient cells. However, the main target of this approach is to either create artificial vesicles with anticancer drugs as cargo for better, more targetable, chemotherapeutic distribution, modify cells to pack specified molecules (siRNA, lncRNA, etc.) to the targeted cells, or sensitize them to currently inefficient therapy (Jang et al., 2013; Wang P. P. et al., 2019). Many efforts are also taken to analyze the effect and mechanism of action of mesenchymal stem cells (MSCs) released sEVs on cancer cells. The mechanism of MSC-derived sEVs is controversial and considered to be contradictory. From one way, there are much data showing enhancement of proliferation of cancer cells, increased angiogenesis, and metastasis by MSC-derived sEVs. However, other studies show the tumor suppression activity of MSC-derived sEVs and great potential for manipulating the cargo they are carrying. Despite many efforts, MSC-derived sEVs, still are very controversial and require more knowledge before proper clinical application in cancer treatment (Vakhshiteh et al., 2019).

**TABLE 4:** Examples of biomarkers with both diagnostic and therapeutic potential in UTC. BCa–urinary bladder cancer, UTC–urinary tract carcinomas, PCa–prostate cancer, RCC–renal cell carcinoma, EMT–epithelial–mesenchymal transition, sEVs–small extracellular vesicles.

Biomarker	Type of UTC	Source of sEV	Potential dual-use in treatment and diagnosis of UTC	Reference
miR-888	PCa	Urine	**Treatment target**–overexpression increases proliferation, migration, and colony formation of PCa cells	Hasegawa et al. (2018)
			Diagnostic target–a higher level of mir-888 in urine sEVs was found only in patients with high-grade PCa	
circ_0044516	PCa	Blood	**Treatment target**–suppression of circ_0044516 resulted in lower proliferation and migration rate in PCa cells	Li et al. (2020)
			Diagnostic target–higher levels of circ_0044516 were found in high-grade PCa patients	
αvβ3 integrin	PCa	Blood	Treatment target–αvβ3 integrin is taking part in adhesion, invasion, immune escape, and neovascularization of tumor cells	Krishn et al. (2019)
			Diagnostic target–selectively present on PSMA-positive sEVs from PCa patients	
lncRNA-UCA1	BCa	Blood	**Treatment target**–sEVs containing lncRNA-UCA1 released from cancer cells under hypoxic conditions promote cancer progression through EMT	Xue et al. (2017)
			Diagnostic target–lncRNA-UCA1 level in sEVs from BCa patients is significantly higher	
lncPTENP1	BCa	Blood	**Treatment measure**–sEVs containing lncPTENP1 are acting as bladder cancer cell suppressors, attenuating tumor growth	Zheng et al. (2018)
			**Diagnostic target–**lncPTENP1 level in sEVs of high-grade BCa patients is significantly lower compared to low grade and healthy control	
miR-633b	BCa	Blood	**Treatment target**–miR-633b carried by sEVs increased proliferation and epithelial–mesenchymal transition	Yin et al. (2020)
			Diagnostic target | miR-633b level in sEVs from BCa patients was significantly higher than that in healthy control	
miR-4644	BCa	Blood	**Treatment target**–in the mouse model, inhibition of miR4664 resulted in suppression of BCa tumorigenesis	Yan et al. (2020)
			**Diagnostic target–**miR-4644 level in sEVs from BCa is significantly higher than that in healthy patients	
miR-224	RCC	Blood	**Treatment target**–overexpression of miR-224 caused increased proliferation and migration with a lower apoptosis rate in RCC cells	Fujii et al., (2017)
			**Diagnostic target**–high level of miR-224 in sEVs is correlated with lower cancer-specific survival rate in RCC patients	
miR-19b-3p	RCC	Blood	**Treatment target** | in *vitro* study, it was shown that miR-19b-3p carried by CD103 + sEVs enhance migration and EMT	Wang et al. (2019b)
			**Diagnostic target–**the increased percentage of CD103 + sEVs carrying miR-19b-3p was increased in metastatic RCC patients compared to non-metastatic	

Much work is still required to define standards and raise awareness of the proper quality of analysis methods among researchers, such as the Guidelines of the International Society for Extracellular Vesicles and taskforces for specific applications. However, during the screening procedure for this study, many studies have been disqualified because of the lack of at least one method for the size and concentration analysis of purified vesicles. Not a small number of such studies show that proper methodology implementation in EV studies is not yet established. Another often occurring problem is the lack of or feeble description of EV purification/separation methods, which can significantly influence results obtained by researchers. There is a disproportion between clinical and biological approaches for obtained result interpretation. From a clinical-diagnostical point of view, even if the selected method does incorporate any uncertainty, whether sEVs carry the biomarker, or another part of biofluids, such as RBPs or freely dispersed proteins are the case, it does not matter, as long as it gives clinically essential data and allows proper diagnosis. From the other point, a proper understanding of analyzed markers’ biogenesis is necessary to take advantage and propose new prophylactic or therapeutic options. The sEVs are an inseparable part of cancer cells. The research analyzing them will undoubtedly bring even more understanding of the pathomechanism of cancer and new diagnostic and therapeutic targets. The relative ease of obtaining biofluids rich in sEVs (blood/urine) makes sEVs an essential part of the liquid biopsy approach for UTC diagnosis and has already led to the creation of commercially available tests such as ExoDx™ Prostate EPI-CE (Exosome Diagnostics GmbH; Germany).
